# The FilZ Protein Contains a Single PilZ Domain and Facilitates the Swarming Motility of *Pseudoalteromonas* sp. SM9913

**DOI:** 10.3390/microorganisms11061566

**Published:** 2023-06-13

**Authors:** Qi Sheng, Ang Liu, Peiling Yang, Zhuowei Chen, Peng Wang, Haining Sun, Chunyang Li, Andrew McMinn, Yin Chen, Yuzhong Zhang, Hainan Su, Xiulan Chen, Yuqiang Zhang

**Affiliations:** 1 State Key Laboratory of Microbial Technology, Shandong University, Qingdao 266237, China; 2 Frontiers Science Center for Deep Ocean Multispheres and Earth System, College of Marine Life Sciences, Ocean University of China, Qingdao 266100, China; 3 Laboratory for Marine Biology and Biotechnology, Pilot National Laboratory for Marine Science and Technology, Qingdao 266237, China; 4 Institute for Marine and Antarctic Studies, University of Tasmania, Hobart, TAS 7005, Australia; 5 School of Life Sciences, University of Warwick, Coventry CV4 7AL, UK

**Keywords:** marine sedimentary bacteria, single-PilZ-domain protein FilZ, c-di-GMP, bacterial flagella, FilZ-mediated swarming motility

## Abstract

Swarming regulation is complicated in flagellated bacteria, especially those possessing dual flagellar systems. It remains unclear whether and how the movement of the constitutive polar flagellum is regulated during swarming motility of these bacteria. Here, we report the downregulation of polar flagellar motility by the c-di-GMP effector FilZ in the marine sedimentary bacterium *Pseudoalteromonas* sp. SM9913. Strain SM9913 possesses two flagellar systems, and *filZ* is located in the lateral flagellar gene cluster. The function of FilZ is negatively controlled by intracellular c-di-GMP. Swarming in strain SM9913 consists of three periods. Deletion and overexpression of *filZ* revealed that, during the period when strain SM9913 expands quickly, FilZ facilitates swarming. In vitro pull-down and bacterial two-hybrid assays suggested that, in the absence of c-di-GMP, FilZ interacts with the CheW homolog A2230, which may be involved in the chemotactic signal transduction pathway to the polar flagellar motor protein FliM_p_, to interfere with polar flagellar motility. When bound to c-di-GMP, FilZ loses its ability to interact with A2230. Bioinformatic investigation indicated that *filZ*-like genes are present in many bacteria with dual flagellar systems. Our findings demonstrate a novel mode of regulation of bacterial swarming motility.

## 1. Introduction

Motility is important for bacterial nutrient assimilation, growth, virulence, biofilm formation, and cell–cell contact [1]. Among various motility modes, swimming and swarming are vital in flagellated bacteria. Swimming is the movement in aqueous environments that is driven by one or several rotating flagella, with individual cells in motion [2,3,4], while swarming is the flagella-driven rapid movement of a large number of bacterial cells on viscous surfaces [3,5,6,7]. Flagella are sophisticated nanomachines [8,9]. The number and arrangement of flagella vary among bacterial species. Some bacteria possess both constitutive polar flagella and inducible lateral flagella to support swimming and swarming motilities, respectively [8,9,10]. There is some evidence for interactions between the two flagellar systems. In the deep-sea bacterium *Shewanella piezotolerans* WP3, mutations interfering with the function of the polar flagellum induce the expression of lateral flagellar genes, while mutations disrupting lateral flagellar genes result in a decrease in the transcription of polar flagellar genes [11]. In *Vibrio parahaemolyticus*, the production of lateral flagella is inhibited by the expression of the polar flagellar genes and activated by the expression of the LafK regulator of lateral flagellar genes [12,13,14]. Despite these studies, it is still unclear whether and how the movement of the polar flagellum is controlled during the swarming of bacteria that possess both flagellar systems.

Bacterial swarming can be directly or indirectly controlled by intracellular c-di-GMP concentrations [15]. c-di-GMP affects bacterial swarming motility by binding diverse protein effectors. Among these, proteins having single or multiple PilZ domains are prevalent [16,17]. The functions of several proteins with PilZ domains are positively regulated by c-di-GMP. Among multi-domain PilZ proteins, YcgR in *Escherichia coli* and MotI in *Bacillus subtilis* suppress swarming motility by interacting with the stator protein MotA [18,19]. FlgZ in *Pseudomonas aeruginosa* PA14 interacts with another stator protein, MotC, to suppress swarming motility [20], and PlzD in *Vibrio alginolyticus* inhibits swarming motility in an unknown manner [21]. Among single-PilZ-domain proteins, MotL from the lateral flagellar gene cluster of *Shewanella putrefaciens* specifically interferes with the motor of the lateral flagella to reduce swarming motility [22]. So far, no single-PilZ-domain protein has been reported to facilitate bacterial swarming.

Deep-sea sediment is a special and poorly characterized microbially driven habitat [23]. To adapt to the extreme conditions of most deep-sea sediments, sedimentary bacteria evolve various special features [23,24,25]. The deep-sea sedimentary bacterium *Pseudoalteromonas* sp. SM9913 has dual flagellar systems, which are encoded by two independent gene clusters. The constitutive polar flagellum is essential for swimming, and the induced lateral flagella are essential for swarming [23,26]. The function of the polar flagellum is mediated by the chemotaxis system. A complete complement of the chemotaxis signaling system exists in the polar flagellar gene cluster, including the genes *cheW*, *cheA*, *cheB*, *cheZ*, *cheY*, *cheR*, and *cheV* [23]. Because of a nonsense mutation in the flagellin gene of the polar flagellar gene cluster, strain SM9913 has an abnormally short polar flagellum compared to other *Pseudoalteromonas* strains. The short polar flagellum has only a small effect on strain SM9913 swimming [27]. Even so, it should be borne in mind that cells with a normal-length polar flagellum might behave somewhat differently than cells of the SM9913 strain used in this study.

This study investigates the regulation of polar-flagellum-driven motility during swarming of strain SM9913. We identified an uncharacterized gene, *filZ* (*PSM_RS04475*), located in the lateral flagellar gene cluster, and analyzed the function of protein FilZ encoded by gene *filZ* in SM9913 swarming. The results indicate that FilZ interferes with polar flagellar motility to facilitate swarming via a chemotaxis signal transduction pathway consisting of proteins A2230 (CheW), A2236 (CheA), and A2238 (CheY) that is directed at protein FliM_p_ of the polar flagellar motor.

## 2. Materials and Methods

### 2.1. Strains, Plasmids, and Growth Conditions

Strain SM9913 was grown as previously described [27]. The plasmids pET-22b (+) and pGEX-4T-1 (Novagen, Darmstadt, Germany) and strains *E. coli* DH5α and *E. coli* BL21 (DE3) were used for gene cloning and protein overexpression, respectively. Strain *E. coli* MW3064 was used for in-frame deletion. *E. coli* DH5α and *E. coli* BL21 (DE3) were grown as previously described [27]. When needed, ampicillin and kanamycin were used at 100 µg/mL and 50 µg/mL, respectively. Strains and plasmids are listed in Table S1.

### 2.2. Gene Cloning, Mutation, and Protein Overexpression

Genes *PSM_A2230* and *filZ* were cloned from the genomic DNA of strain SM9913 and ligated with plasmid pET-22b (+) to generate pET-22b-*A2230* and pET-22b-*filZ*. Gene *filZ* was also ligated with vector pGEX-4T-1 to generate pGEX4T-1-*filZ*. All of the site-directed mutations in FilZ were introduced with the QuickChange II mutagenesis kit (Agilent, Santa Clara, CA, USA) and verified by DNA sequencing. The verified vectors were introduced into *E. coli* BL21 (DE3). The recombinant strains were cultured at 37 °C for 4–5 h in LB medium containing 100 µg/mL ampicillin. When the OD_600_ of the cultures reached 0.8, 0.1 mM isopropyl-β-D-thiogalactopyranoside (IPTG) was added to induce protein expression, and the cells were then incubated at 16 °C for 20 h.

### 2.3. Protein Purification

The recombinant cells were pelleted (10,000× *g* for 10 min at 15 °C), resuspended in 40 mM phosphate buffer (pH 7.5) supplemented with 100 mM NaCl, and then disrupted with passage through a JN-02C French press (JNBIO, Guangzhou, China) at 300 psi. After centrifugation (15,000× *g* for 10 min at 15 °C), the pellets were exposed to 1% Triton and dissolved in PGE buffer (40 mM phosphate buffer [pH 7.5], supplemented with 50 mM NaCl, 5% glycerol, 0.5 mM EDTA, and 2 mM dithiothreitol) containing 6 M guanidine hydrochloride (GndHCl). The proteins were then dialyzed at 4 °C against PGE buffer. The soluble proteins thus obtained were further purified by chromatography as previously described [28].

### 2.4. Isothermal Titration Calorimetry

Measurements of the affinities and stoichiometries of FilZ and its mutant variants with c-di-GMP were performed with a MicroCal iTC200 system (GE Healthcare, Danderyd, Sweden) as previously described [29,30]. Data were processed using the MicroCal ORIGIN version 7.0 software.

### 2.5. Construction of the Mutants of Strain SM9913

The deletion mutant strains *(*Δ*filZ*, Δ*2230*, and Δ*0915)* were constructed as described by Sheng et al. [27] with some modifications. Pairs of primers (pK18-*filZ*-up-F/R and pK18-*filZ*-down-F/R; pK18-*2230*-up-F/R and pK18-*2230*-down-F/R; and pK18-*0915*-up-F/R and pK18-*0915*-down-F/R) were synthesized to amplify the homologous arms of the *filZ*, *PSM_A2230*, and *PSM_A0915* genes separately. The amplified genes were then ligated into the suicide vector pK18*mobsacB*-Ery. The plasmids thus constructed were transferred into strain SM9913 for further in-frame deletion. The target sequences were confirmed by PCR with the primer pairs (*filZ*-screen-F/R, *2230*-screen-F/R, and *0915*-screen-F/R). All the primers are listed in Table S2.

### 2.6. Construction of Complementary Strains of SM9913

The complementary strains of SM9913 were constructed as previously described [26,27,31,32]. The plasmid pEV was used as a shuttle vector. All plasmids overexpressed the cloned proteins under the control of the plasmid-encoded promoter. Primers pEV-*filZ*-F/R were used to construct the plasmid pEV*^filZ^*. To construct the plasmid pEV*^filZ-R13A^*, site-directed mutagenesis was performed using primers *filZ*-*R13A*-F/R and the template pEV*^filZ^*. The resulting plasmids were introduced into the corresponding mutants.

### 2.7. Bacterial Motility Assay

The motility of strain SM9913 and its mutant derivatives was assayed as previously described [26]. Briefly, marine LB plates with 0.3% or 0.5% agar (Bacto^TM^ agar, Franklin Lakes, NJ, USA) were used to assay swimming or swarming, respectively. Plates were incubated at 15 °C, and the colony diameters on the swarm plates were measured over 3 days. The scatter plots of the changes in colony diameter were made using OriginPro 8.5 software. The scatterplots were then fit nonlinearly using the Boltzmann function in the Growth/Sigmoidal class. The curves of the colony expansion rates were obtained by plotting differences in diameter as a function of time from 24 to 72 h, using the fitted curves.

### 2.8. Total RNA Extraction and RT-qPCR

The transcription levels of *PSM_A2230*, *filZ*, and all the other genes from the lateral flagellar gene cluster were determined by Real-Time quantitative PCR (RT-qPCR). A 5 μL aliquot of an overnight culture of strain SM9913 was spotted at the center of each 0.5% agar plate, and the plates were incubated at 15 °C. Cells were collected from the colony edge at different culture times. The extraction of total RNA, the synthesis of cDNA, and the qPCR reaction were performed as described previously [27]. The relative levels of expression of *filZ* and *PSM_A2230* were normalized using *rpoD* (the RNA polymerase sigma factor gene) as an internal reference.

### 2.9. Atomic Force Microscopy Imaging

SM9913 cells were collected from the edge of the swarming colonies at different culture times, and atomic force microscopy imaging was performed as previously described [27].

### 2.10. The Construction and Screening of E. coli BACTH Library and Two-Hybrid Assays

The construction of the *E. coli* BACTH (Bacterial Acetylate Cyclase Two-Hybrid) library and the library screening were performed as previously described with slight modifications [33,34]. Plasmids pKT25 and pUT18C were gifts from Professor Xiaoxue Wang [35]. Fragments of the *Sau*3AI-digested SM9913 genomic DNA were ligated with the *Bam*HI-digested pKT25 vector, and aliquots of the ligation mixture were introduced chemically into 100 μL competent *E. coli* DH5α cells (Tsingke Biotechnology Co., Beijing, China). The ligation mixture was mixed with the thawed competent cells, and the cells were incubated on ice for 30 min. Cells were then incubated at 42 °C for 45 s, followed by incubation on ice for 2 min. After incubation, 0.5 mL of LB medium without antibiotics was added, and cells were preincubated at 37 °C for 1 h. Cells were then plated onto LB agar plates containing 50 μg/mL kanamycin, and the plates were incubated at 30 °C for 36 h. All the colonies from the plates were then collected, and the purified plasmids were used for the BACTH plasmid DNA library.

For library screening, protein FilZ-R13A was used as the bait [33,34]. Plasmid pUT18C-*filZ-R13A* (encoding the T18-FilZ-R13A hybrid protein) was introduced into the *E. coli* host strain BTH101. The resultant strain, BTH101 (pUT18C-*filZ-R13A*), was then transformed with the SM9913 DNA library, using 100 ng DNA. The transformed cells were plated on MacConkey medium (Solarbio, Beijing, China) until the red colonies became large enough to isolate. The plasmids from the isolates were sequenced to verify that they contained inserted DNA.

### 2.11. Bacterial Two-Hybrid Assays

The bacterial two-hybrid assay was performed using the BACTH system [35] with slight modifications. Plasmid pKT25 was digested with *Kpn*I, and pUT18C was digested with *Bam*HI and *EcoR*I. The coding region of the target genes (including the stop codon) was ligated into the digested plasmids. The recombinant pKT25 and pUT18C plasmids were introduced into *E. coli* BTH101 for the two-hybrid assays.

### 2.12. Extraction and Detection of c-di-GMP

Strain SM9913 cells at the edge of the swarming colony were collected after incubation for 48, 54, 60, 66, and 72 h. c-di-GMP was extracted from each sample as described by Petrova et al. [36]. The c-di-GMP content was analyzed on a carbon-18 reversed column (Waters, Drinagh, Ireland) using high-performance liquid chromatography (LC-20AD, Shimadzu, Kyoto, Japan) with commercial c-di-GMP (Biolog, Hayward, CA, USA) as a standard. The mobile phases were as follows: solution A, 10 mM ammonium acetate aqueous solution; solution B, 10 mM ammonium acetate methanol solution. The complete separation procedure lasted 40 min: 0–9 min, 99% solution A, 1% solution B; 9–14 min, 85% solution A, 15% solution B; 14–19 min, 75% solution A, 25% solution B; 19–26 min, 10% solution A, 90% solution B; 26–40 min, 1% solution A, 99% solution B. Based on the standard curve with c-di-GMP, the c-di-GMP content in each sample, (T_c-di-GMP_), was calculated.

### 2.13. Pull-Down Experiment

The pull-down experiment was performed as described by Sun et al. [37] with some modifications. Glutathione S-transferase (GST)-tagged FilZ, GST-tagged FilZ-R13A, and the His-tagged A2230 proteins were expressed in *E. coli* BL21 (DE3) and purified. For the interaction of FilZ (or FilZ-R13A) with A2230, the 2 proteins (0.5 mg each) were mixed and incubated at 4 °C for 1 h. For the interaction of FilZ-c-di-GMP with A2230, 0.5 mg FilZ was incubated with a 10 μM c-di-GMP solution at 4 °C for 1 h. Then, 0.5 mg A2230 was added, followed by further incubation at 4 °C for 1 h. For the interaction of the FilZ-A2230 complex with c-di-GMP, a mixture of 0.5 mg FilZ and 0.5 mg A2230 was incubated at 4 °C for 1 h. Then, 10 μM c-di-GMP was added, followed by further incubation at 4 °C for 1 h. After incubation, the samples were added to a glutathione sepharose 4B (GE healthcare, Amersham, UK) column pre-treated with 40 mM phosphate buffer (pH 7.5) containing 100 mM NaCl. The proteins were eluted with the phosphate buffer containing 20 mM reduced glutathione. The eluted proteins were detected by immunoblotting.

### 2.14. Immunoblot

The proteins FilZ and His-tagged A2230 were separated by SDS-PAGE, and the His-tagged A2230 in the electrophoresis gel was transferred (350 mA, 150 min) to the ECL membrane for immunoblotting as described previously [38,39]. The membrane was incubated with TBST buffer containing the primary antibody (Anti 6× His tag antibody, Abcam, Cambridge, UK) at 4 °C overnight. After treatment with the secondary antibody (Sheep anti-rabbit IgG H&L, Abcam, UK), the protein bands on the membrane were developed in a myECL imager (ThermoFisher Scientific, Waltham, MA, USA).

### 2.15. Chemotaxis Assays

Chemotaxis of strain SM9913 and its gene *PSM_A2230* deleted derivative were tested by capillary assay with some modifications [40]. Sucrose (1.0 g/L) or casein (2.0 g/L) was used as a chemoattractant. Glucose (2.0 g/L) was used as the positive control. The cells in the capillaries were incubated at 15 °C for 48–60 h. After 30 min incubation at 15 °C, the cells in the capillaries were serially diluted and plated onto nutrient agar. The plates were incubated at 15 °C for 48–60 h, and the number of colonies was counted. Capillaries containing buffer alone were used as the negative control.

## 3. Results

### 3.1. Effect of Protein FilZ on the Swarming of Strain SM9913

**Figure 1:**
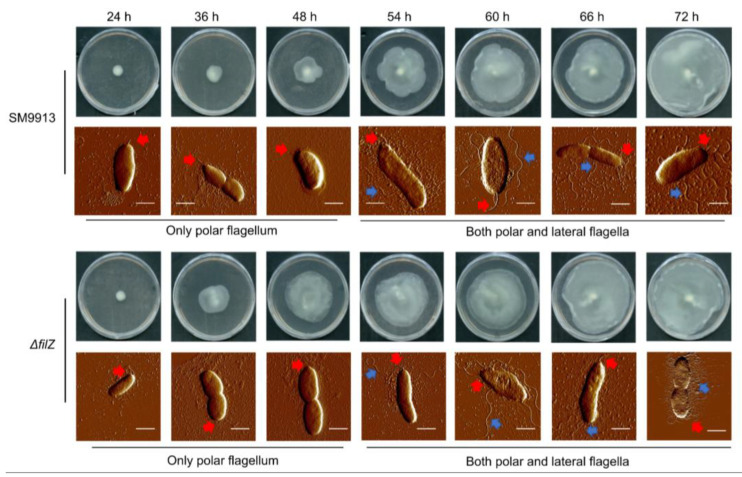
The swarming motility of strain SM9913 and its Δ*filZ* mutant and the production of their flagella. Strains SM9913 and Δ*filZ* were cultured on the swarming plate at 15 °C. The red arrow points to the polar flagellum, and the blue arrow points to the lateral flagella. At least 30 cells were observed at each time point. Bar, 1 μm.

**Figure 2:**
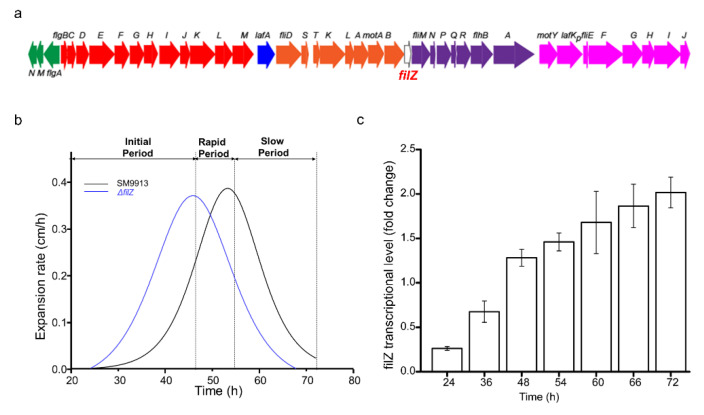
The involvement of FilZ in swarming motility. (**a**) The lateral flagellar gene cluster of strain SM9913 and the location of gene *filZ* within the cluster. (**b**) The colony expansion rates of strain SM9913 and its Δ*filZ* mutant. Swarming of SM9913 was divided into three periods: the initial slow period (0–48 h), the rapid swarming period (48–54 h), and the slow swarming period (54–72 h). In the Δ*filZ* mutant, these periods are advanced by about 5 h. (**c**) The transcription of *filZ* during swarming. Cells used to analyze the transcriptional level were collected from the edge of the swarming colony at different times. The fold change in the transcription of *filZ* was calculated relative to that of the endogenous control gene *rpoD* (the RNA polymerase sigma factor gene). The graph shows data from triplicate experiments (mean ± SD).

To determine the timing of the production of lateral flagella, SM9913 cells were inoculated onto a swarm plate (0.5% agar) from liquid medium. Cells at the edge of the colony were observed with an atomic force microscope from 24 h to 72 h. Most of the observed cells had a polar flagellum at all times. Lateral flagella were observed starting at approximately 50 h (Figure 1 and Figure S1). The swarming process can be divided into three periods: the initial period before the colony expanded (0–48 h); the rapid swarming period, during which the swarming rate kept increasing (48–54 h); and the slow swarming period, during which the swarming rate decreased steadily (54–72 h) (Figure 1 and Figure 2b).

An uncharacterized *filZ* gene is located in the lateral flagellar gene cluster (Figure 2a). The transcription of *filZ* was upregulated during the rapid swarming period (Figure 2c), suggesting that the *filZ* gene product may have a role in swarming motility. To determine how FilZ was involved in swarming, we constructed a Δ*filZ* deletion mutant. The growth in liquid and the temporal order of flagellar production during the swarming process in the Δ*filZ* mutant were similar to those of WT SM9913 (Figure 1 and Figure S2). However, the expansion pattern of the Δ*filZ* colony was quite different. Compared to WT SM9913, the Δ*filZ* colony expanded much more rapidly in the initial period, leading to a precocious swarming phenotype, and then steadily decreased (Figure 1 and Figure 2b). During the rapid swarming period for WT SM9913, the rate of colony expansion of Δ*filZ* steadily decreased, but that of WT SM9913 kept increasing (Figure 2b). These results suggest that the *filZ* gene product facilitates the swarming motility of strain SM9913 in the 48–54 h rapid period.

### 3.2. FilZ Has a Single PilZ Domain That Binds c-di-GMP

**Figure 3:**
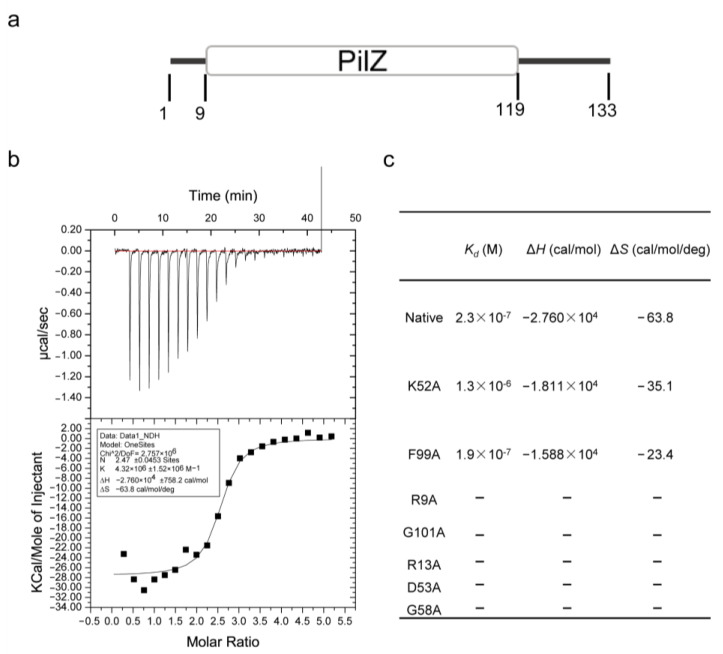
Analyses of the c-di-GMP binding ability of FilZ and identification of the key residues involved in binding c-di-GMP. (**a**) Prediction of the PilZ domain in FilZ. (**b**) Isothermal titration calorimetry (ITC) analysis of c-di-GMP binding to recombinant FilZ. The upper panel shows the raw calorimetric data of the titration. The lower panel shows the corresponding integrated injection heats. The curve represents the best least-squares fit to a one-binding-site model. The graph is representative of three replicates. (**c**) The c-di-GMP binding abilities of the mutant FilZ determined by ITC.

**Figure 4:**
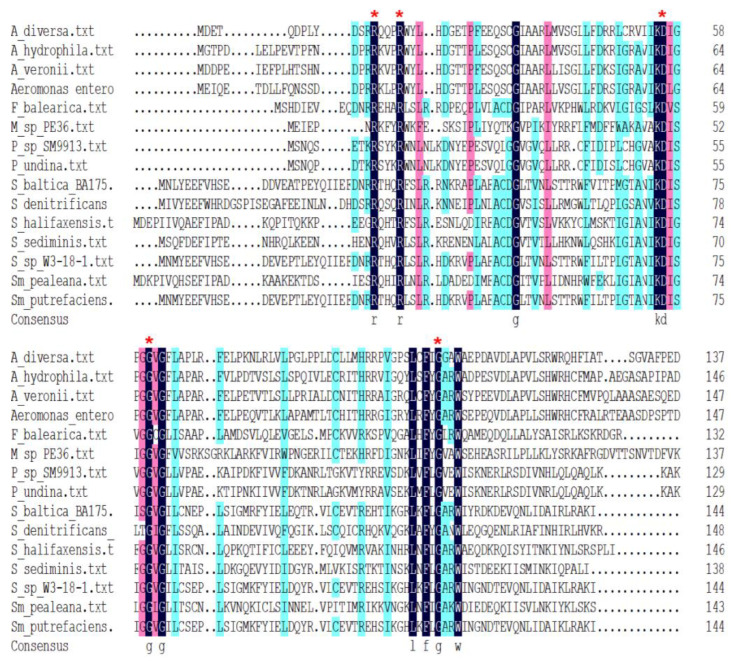
Sequence alignment of FilZ and other c-di-GMP effectors that contain the PilZ domain. The key residues R9, R13, D53, G58, and G101 of FilZ in binding c-di-GMP are highlighted by red asterisks. The most identical residues are highlighted in a black background, followed by a pink and blue background separately.

Sequence analysis indicated that FilZ contains a single PilZ domain (Figure 3a and Figure S3). PilZ domains are known to bind c-di-GMP [17]. We tested the interaction between the recombinant FilZ protein and c-di-GMP using isothermal titration calorimetry (ITC). FilZ displayed a strong c-di-GMP binding ability, with a *K*_d_ value of 230 nM (Figure 3b,c). This property suggests that FilZ may function as a c-di-GMP effector. To determine the key amino acids involved in binding c-di-GMP, we predicted the tertiary structure of FilZ bound to c-di-GMP using I-Tasser. FilZ consists of an N-terminal β-barrel and a flexible C-terminal α-helix, highly similar to the structure of the C-terminal PilZ domain of YcgR from *E. coli* (YcgR-PilZ, PDB ID 5Y6F) (Figure S4). The N-terminal β-barrel contains two motifs, ^9^RXXXR^13^ and ^53^(D/N)XSXXG^58^, that are conserved in PilZ domains [41]. Based on the predicted structure of the complex, c-di-GMP probably interacts with residues R9, R13, K52, D53, G58, F99, and G101 (Figure S4). To determine the importance of these residues in binding c-di-GMP, we used site-directed mutagenesis to insert alanine at all of these positions. Mutants R9A, R13A, D53A, G58A, and G101A all completely lost the ability to bind c-di-GMP (Figure 3c and Figure S5), indicating that R9, R13, D53, G58, and G101 are important for binding c-di-GMP. These residues are conserved in other PilZ-domain proteins (Figure 4). FilZ-R13A was chosen as a representative for further investigation.

### 3.3. FilZ Activity Is Negatively Controlled by c-di-GMP In Vivo

**Figure 5:**
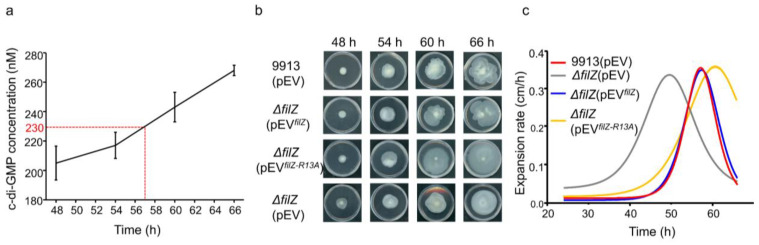
The intracellular c-di-GMP concentration regulates the function of FilZ. (**a**) Intracellular c-di-GMP concentrations during SM9913 swarming. The red dotted line points to the time when intracellular c-di-GMP concentrations reached the *K*_d_ of FilZ (230 nM) for c-di-GMP. The graph shows data from triplicate experiments (mean ± SD). (**b**) Swarming motility at 15 °C. (**c**) The colony expansion rates at 15 °C.

We began by determining the intracellular concentration of c-di-GMP during the swarming process. The average intracellular concentration of c-di-GMP increased continuously (Figure 5a). However, in the rapid swarming period of 48–54 h, the intracellular c-di-GMP concentration was lower than the *K*_d_ value of FilZ for c-di-GMP.

To study the interaction between FilZ and c-di-GMP in vivo, we constructed two complementary strains: Δ*filZ*(pEV*^filZ^*), which has inducible expression of FilZ, and Δ*filZ* (pEV*^filZ-R13A^*) which has inducible expression of FilZ-R13A. The growth of WT SM9913 was decreased by the maintenance of the plasmid pEV (Figures S2 and S6); thus, all the strains containing the pEV plasmid expanded slower on the swarming plates than those without the plasmid. In contrast to strain Δ*filZ*(pEV), both the complementary strains displayed similar increasing swarm expansion rates before 54 h to strain 9913(pEV) (Figure 5b,c). Because the mutant FliZ-R13A had no ability to bind c-di-GMP, this result suggests that FilZ likely functions without binding c-di-GMP in the rapid swarming period. In addition, compared with Δ*filZ*(pEV*^filZ^*) and 9913(pEV), Δ*filZ*(pEV*^filZ-R13A^*) showed a slower decline in swarming rate in the slow swarming period, in which the intracellular c-di-GMP concentrations were higher than the *K*_d_ of FilZ for c-di-GMP (Figure 5b,c). Taken together, these results suggest that FilZ facilitates the swarming motility of strain SM9913 when it is not bound to c-di-GMP but is inactivated when bound to c-di-GMP. Thus, it seems that higher intracellular c-di-GMP concentrations negatively regulate FilZ function to inhibit swarming.

### 3.4. FilZ Interacts with the CheW-like Protein A2230

**Figure 6:**
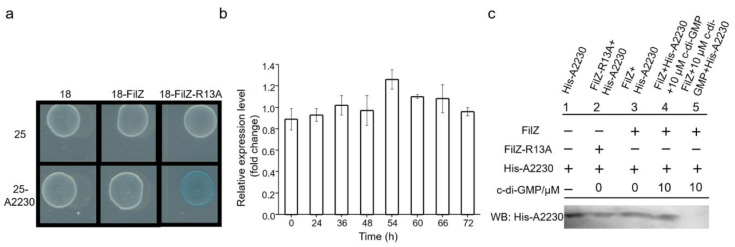
The interaction of FilZ with protein A2230 and the involvement of A2230 in the chemotaxis signal transduction pathway to FliM_p_. (**a**) The protein interacting with FilZ was screened by using a bacterial two-hybrid system (18, pUT18C; 25, pKT25). (**b**) The transcriptional level of gene *PSM_A2230* during swarming. The fold change in the transcriptional level was calculated using *rpoD* (the RNA polymerase sigma factor gene) as an endogenous control. The graph shows data from triplicate experiments (mean ± SD). (**c**) Pull-down assay showing the interaction of FilZ or FilZ-R13A with His-A2230. Each lane shows proteins/c-di-GMP present (+) or absent (−) in the mixture in the assay. The interaction was monitored by determining the amount of His-tagged A2230 with His tag antibody by immunoblotting of each mixture.

To identify the target protein(s) with which FilZ interacted, we performed a screen using a bacterial two-hybrid assay with FilZ-R13A as the bait. Among the proteins expressed in strain SM9913, only A2230 was verified to interact with FilZ-R13A (Figure 6a). Gene *PSM_A2230* is located in the polar flagellar gene cluster. It displayed a stable transcription level during swarming (Figure 6b). Sequence analysis suggested that A2230 is a CheW-like protein, with a high sequence identity (69%) to protein CheW from *P. aeruginosa* (Figure S7). Furthermore, a capillary assay to test the chemotaxis behavior of strain SM9913 and its Δ*2230* mutant showed that the mutant accumulated only 50% as many cells as WT SM9913. Thus, the A2230 protein contributes to chemotaxis mediated by the polar flagellum.

To determine the relationships of c-di-GMP and proteins FilZ and A2230, a pull-down experiment was performed. FilZ interacted with A2230 in the absence of c-di-GMP (Lanes 2 to 4 in Figure 6c); however, upon binding c-di-GMP, FilZ could no longer interact with A2230 (Lane 5 in Figure 6c and Figure S8). Thus, in SM9913 swarming cells, FilZ likely exerts its functions through interacting with A2230 when not binding c-di-GMP.

**Figure 7:**
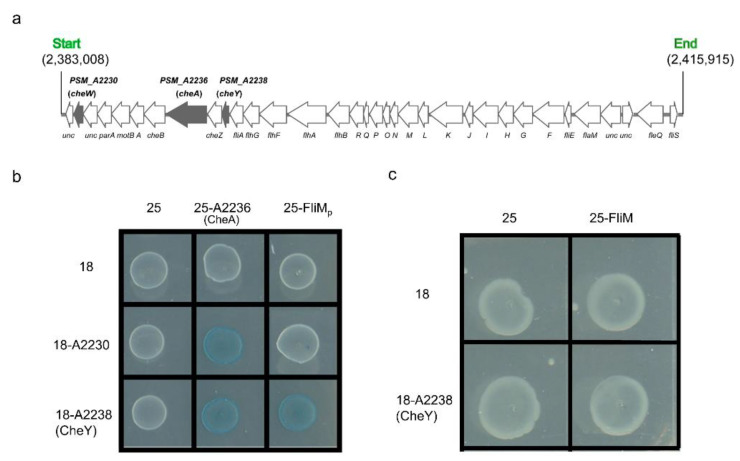
Chemotaxis signal transduction pathway to the polar flagellum in strain SM9913. (**a**) Part of the polar flagellar gene cluster of strain SM9913. Genes *PSM_A2230* encoding a CheW homolog, *PSM_A2236* encoding a CheA protein, and *PSM_A2238* encoding a CheY protein are shown in grey. (**b**) Identification of the chemotaxis signal transduction pathway involving A2230, A2236, and A2238, and the target polar flagellar motor protein FliM_p_ (18, pUT18C; 25, pKT25). The blue color indicates an interaction between A2230 (CheW) and A2236 (CheA), as well as an interaction between A2238 (CheY) and both A2236 (CheA) and FliM_p_. (**c**) Identification of the interaction between A2238 (CheY) and the lateral flagellar motor protein FliM. The white color indicates that A2238 (CheY) does not interact with FliM.

Previous work has shown that CheW is part of the flagella-mediated chemotaxis signal transduction system that consists of the chemotaxis proteins CheW, CheA, and CheY. Phosphorylated CheY acts on the flagellar motor protein FliM to regulate flagellar motility [42,43]. Sequence analysis showed that genes *PSM_A2236* and *PSM_A2238* located in the polar flagellar gene cluster encode proteins CheA and CheY, respectively (Figure 7a). To determine whether the CheW/CheA/CheY signal transduction pathway interacts with the polar flagellar motor protein FliM_p_ or the lateral flagellar motor protein FliM, we performed two-hybrid experiments using five reporter plasmids: pKT25-*cheA*, pKT25-*fliM*, pKT25-*fliM_p_*, pUT18C-*A2230*, and pUT18C-*cheY*. The results show that protein A2230 (CheW) interacted with A2236 (CheA), and that protein A2238 (CheY) interacted with A2236 and FliM_p_. Thus, all three proteins may be involved in a chemotaxis signaling pathway directed at FliM_p_ rather than FliM (Figure 7b,c). Together with the finding that FilZ interacted with A2230, it seems likely that FilZ influences swarming indirectly through an effect on polar flagellar motility mediated by the chemotaxis signaling pathway.

### 3.5. FilZ Interferes with the Polar Flagellar Motility

**Figure 8:**
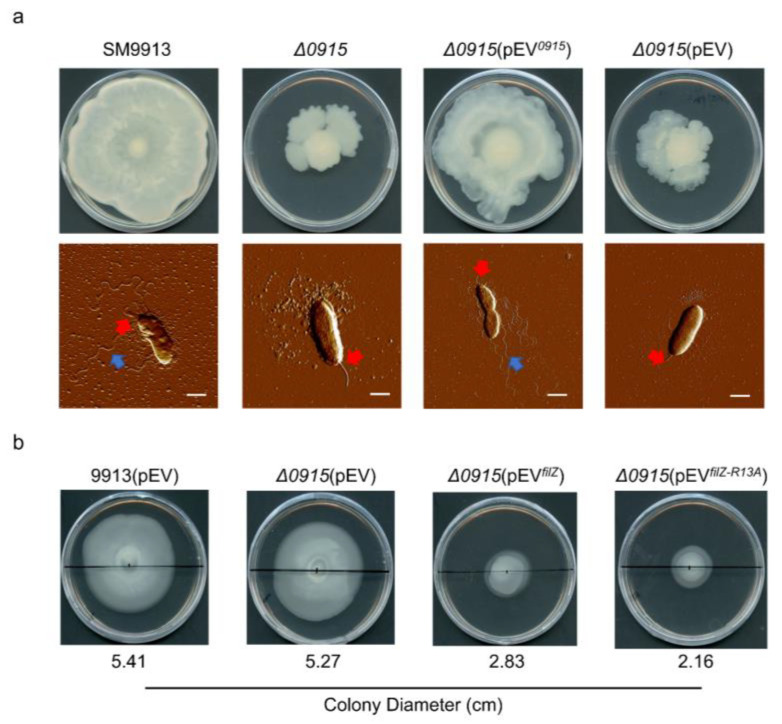
Analysis of the effect of FilZ on polar flagellum motility. (**a**) Swarming motility and flagellation of strains SM9913, Δ*0915*, Δ*0915*(pEV*^0915^*), and Δ*0915*(pEV) on the swarming plates at 15 °C for 68 h. At least 30 cells were observed for the flagellation at each time point. The red arrow points to the polar flagellum, and the blue arrow to the lateral flagella. Bar, 1 μm. (**b**) Colonies formed by strains 9913(pEV), Δ*0915*(pEV)*,* Δ*0915*(pEV*^filZ^*), and Δ*0915*(pEV*^filZ-R13A^*) in 0.3% soft agar. Plates were incubated at 15 °C for 68 h.

To explore the effect of FilZ on polar flagellar motility further, we constructed the mutant Δ*0915*, which could not produce the lateral flagella. Protein A0915 is a homolog of LafK, which is the master regulator of the expression of lateral flagella in *Vibrio parahaemolyticus* (Figure S9) [14]. As expected, protein A0915 was needed for the expression of most of the genes in the lateral flagellar cluster, and the Δ*0915* mutant produced only a polar flagellum and lost swarming motility (Figure 8a). We also constructed strain Δ*0915*(pEV*^filZ^*) and strain Δ*0915*(pEV*^filZ-R13A^*) and observed their motility in soft (0.3%) agar. Compared with strains SM9913 (pEV) and Δ*0915*(pEV), which formed similar spreading colonies, both strains Δ*0915*(pEV*^filZ^*) and Δ*0915*(pEV*^filZ-R13A^*) showed impaired spreading (Figure 8b). This result suggests that FilZ interferes with the polar flagellum when it is not bound to c-di-GMP. Presumably, expression of the chromosomal *filZ* gene is not induced in the free-swimming SM9913 (pEV) and Δ*0915*(pEV) cells.

### 3.6. The Phylogenetic Distribution of FilZ

**Figure 9:**
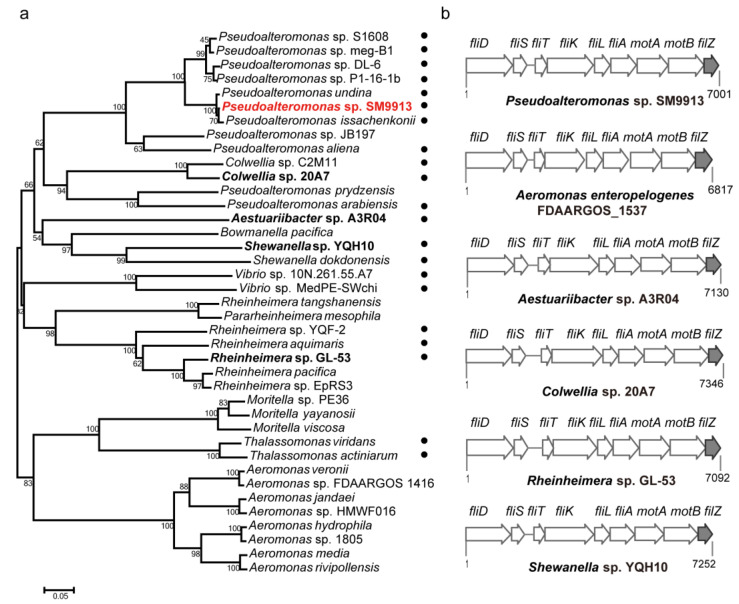
The phylogenetic distribution of FilZ. (**a**) Phylogenetic analysis of FilZ homologs. Strain SM9913 is highlighted in red font. Marine bacteria are indicated by dots. Other marine bacteria in (**b**) are highlighted in bold font. Information about all of the bacterial species can be found in Table S3. (**b**) Alignment of the lateral flagellar gene clusters in γ-proteobacteria containing the *filZ* gene. Except for the strain *Aeromonas enteropelogenes* FDAARGOS_1537, all are marine bacteria. The *filZ* gene lies within the lateral flagellar gene cluster and is located adjacent to the *motAB* genes.

To learn how prevalent this mode of regulation of swarming is among bacteria, we searched the non-redundant protein database in NCBI. Through sequence alignment and phylogenetic relationship analysis, we found that all bacterial species with FilZ homologs belong to the γ-proteobacteria and that more than half are marine bacteria (Figure 9a and Table S3). Furthermore, part of the lateral flagellar gene cluster, including *filZ* and another five or six adjacent genes, was present in many marine bacteria possessing dual flagellar systems. These include the genera *Pseudoalteromonas*, *Aeromonas*, *Shewanella*, and others (Figure 9b). Thus, FilZ-like modulation of swarming may be a common strategy adopted by benthic marine bacteria.

## 4. Discussion

In this study, FilZ, a protein encoded by the *filZ* gene in the lateral flagellar gene cluster of strain SM9913, was shown to be a c-di-GMP effector that facilitated swarming motility. FilZ consists largely of a single PilZ domain that is responsible for binding c-di-GMP. When not bound to c-di-GMP, FilZ interacted with the CheW homolog A2230, which was part of the chemotaxis signal transduction pathway for the polar flagellum. The R13A variant of FilZ, which could not bind c-di-GMP and thus was able to bind A2230 even in the presence of high levels of c-di-GMP, was constitutively active as a negative regulator of the polar flagellum. Thus, high concentrations of c-di-GMP inhibited swarming because they inactivated a negative regulator of chemotactic control of the polar flagellum. The results indicate that FilZ exerts its function on the abnormally short polar flagellum of the WT SM9913 strain in swarming. In addition, although a previous study showed that the length of the polar flagellum of WT SM9913 has only a small effect on swimming motility [27], the function of FilZ may be different in the strain harboring a normal long polar flagellum, which still awaits further study.

Other PilZ-domain proteins have been reported to be involved in bacterial motility, including YcgR [18], MotI [19], FlgZ [20], PlzD [21], and MotL [22]. These proteins have a negative regulatory effect on swimming or swarming. For example, YcgR in *E. coli* interferes with swarming motility via a “backstop brake” mechanism; after binding c-di-GMP, YcgR interacts with the motor proteins MotA and FliG to reduce the efficiency of torque generation on the peritrichous flagella [18,41]. In contrast to YcgR, which affects the function of both polar and lateral flagella in various species, the single-PilZ-domain protein MotL from the lateral flagellar system of *Shewanella putrefaciens* appears to interact directly with components of the lateral flagellar motors to inhibit the function of lateral flagella when it is bound to c-di-GMP [22]. Compared to these proteins, FilZ has some distinct characteristics. First, its activity is negatively controlled by c-di-GMP. FilZ of strain SM9913 promotes swarming when it is not bound to c-di-GMP. Upon binding c-di-GMP, the function of FilZ may be blocked. Second, although encoded by the lateral flagellar gene cluster, FilZ exerts its effect by interfering with polar flagellar motility to facilitate swarming motility indirectly. This is a direct example of an interplay between the polar and lateral flagellar systems in a bacterium with dual flagellar systems.

Somewhat paradoxically, a Δ*filZ* mutant showed a precocious swarming phenotype. Precocious swarming has also been observed in bacteria with a single flagellar system, such as *Proteus mirabilis* [44,45], *Salmonella enterica* serovar Typhimurium [46], and *Serratia marcescens* [47,48,49,50]. This phenotype is mediated by the master flagellar regulator FlhDC. However, little is known concerning the regulation of the precocious swarming phenotype in bacteria with dual flagellar systems. Sequence analysis suggested that there are no FlhDC homologs in strain SM9913. Thus, we assume that the precocious swarming phenotype of the Δ*filZ* mutant may have a very different cause. As FilZ interfered with function of the polar flagellum in strain SM9913, the precocious swarming phenotype of Δ*filZ* may be driven by the unaffected polar flagellum. Clearly, the mechanism underlying precocious swarming in strain SM9913 requires further study.

Chemotaxis signal transduction has been well-studied in many flagellated bacteria. It influences both swimming and swarming motilities by regulating the direction of flagellar rotation [5,51]. The CheA histidine kinase and the CheY regulator of flagellar switching are central to chemotaxis signal transduction. When coupled with CheW and a chemoreceptor, CheA is activated and phosphorylates CheY. For polar flagella, which can rotate both clockwise and counter-clockwise, phospho-CheY interacts with the FliM protein in the flagellar motor to induce clockwise flagellar rotation, leading to a change in the swimming direction [52,53]. For lateral flagella, which only rotate counter-clockwise, especially from bacteria possessing dual flagellar systems such as *V. alginolyticus*, this binding slows down the lateral flagella-driven motility [53]. In strain SM9913, we found a complete set of genes encoding the chemotaxis signaling system in the polar flagellar gene cluster, including genes *PSM_A2230* (*cheW*), *PSM_A2236* (*cheA*), and *PSM_A2238* (*cheY*). Pull-down assays demonstrated that proteins FilZ and A2230 interacted, and two-hybrid assays established the link between the proteins A2230, A2236, and A2238 and the FliM_p_ protein of the polar flagellar motor. These results suggest that FilZ may affect the polar flagellum via this chemotaxis signal pathway to promote swarming in strain SM9913. The negative effect of FliZ on the function of the polar flagellum was demonstrated by the impaired spreading of cells with overproduced, plasmid-encoded FilZ or FilZ-R13A in 0.3% agar. The cells move through this agar by swimming driven by the polar flagellum. Cells that cannot reverse the direction of flagellar rotation are defective in chemotaxis and therefore not impaired in spreading [53]. Wild-type cells swam under these conditions because expression of the chromosomal *filZ* gene was not induced.

In summary, the previously uncharacterized FilZ protein, which has a single PilZ domain that binds c-di-GMP, was found to facilitate surface motility in the rapid swarming period of the deep-sea sedimentary bacterium *Pseudoalteromonas* sp. SM9913. FilZ binds to the CheW homolog A2230, thereby impairing the function of the polar flagellum. FilZ is unable to bind A2230 when it is bound to c-di-GMP, meaning that c-d-GMP is a negative regulator that inhibits swarming indirectly. FilZ is encoded by a gene in the cluster that encodes the components of the lateral flagella that propel surface swarming. Our study identifies a previously unknown interplay between the polar and lateral flagella and provides new insights into the regulation of motility in bacteria with dual flagellar systems.

## Data Availability

All data are available in the main text or the Supplementary Materials. Protein FilZ has been deposited in the NCBI BioProject database under accession number WP_013464393.1.

## Supplementary Materials

The following supporting information can be downloaded at https://www.mdpi.com/article/10.3390/microorganisms11061566/s1. Table S1: Bacterial strains and plasmids used in this study; Table S2: Primers used in this study; Table S3: Information of bacteria involved in the phylogenetic analysis of FilZ homologs and the alignment of the lateral flagellar cluster regions containing the *filZ* gene; Figure S1: Observation of the production of flagella during strain SM9913 swarming at 50 h; Figure S2: Growth curves of strain SM9913 and its mutant Δ*filZ*; Figure S3: Phylogenetic analysis of bacterial PilZ domains; Figure S4: Predicted structure of protein FilZ complexed with the c-di-GMP molecule; Figure S5: ITC detection of the c-di-GMP binding ability of recombinant FilZ mutants including FilZ-R9A, FilZ-R13A, FilZ-K52A, FilZ-D53A, FilZ-G58A, FilZ-F99A and FilZ-G101A; Figure S6: Growth curves of strains 9913(pEV), Δ*filZ*(pEV*^filZ^*), Δ*filZ*(pEV*^filZ-R13A^*) and Δ*filZ*(pEV); Figure S7: Sequence analysis of protein A2230; Figure S8: Pull down assay of the interaction between FilZ or FilZ-R13A and His-A2230 in vitro combined with Western Blot; Figure S9: Sequence analysis of protein A0915. References [26,35,38,54] are cited in the Supplementary Materials.
