# Supplementary material for: The FilZ Protein Contains a Single PilZ Domain and Facilitates the Swarming Motility of Pseudoalteromonas sp. SM9913
Source: Microorganisms. 2023 Jun 13;11(6):1566. doi: 10.3390/microorganisms11061566 (PMC10304486; doi:10.3390/microorganisms11061566)
Supplement: Supplementary file 1 [file microorganisms-11-01566-s001.zip › microorganisms-2364150-supplementary.pdf]

Supplemental Material

The FilZ Protein Contains a Single PilZ Domain and Facilitates the Swarming Motility of *Pseudoalteromonas* sp. SM9913

Qi Sheng [1], Ang Liu [1], Peiling Yang [1], Zhuowei Chen [1], Peng Wang [2,3], Haining Sun [1], Chunyang Li [2,3], Andrew McMinn [2,4], Yin Chen [2,5], Yuzhong Zhang [1,2,3], Hainan Su [1,*], Xiulan Chen [1,3] and Yuqiang Zhang [1,*]

[1] State Key Laboratory of Microbial Technology, Shandong University, Qingdao 266237, China
[2] Frontiers Science Center for Deep Ocean Multispheres and Earth System, College of Marine Life Sciences, Ocean University of China, Qingdao 266100, China
[3] Laboratory for Marine Biology and Biotechnology, Pilot National Laboratory for Marine Science and
Technology, Qingdao 266237, China
[4] Institute for Marine and Antarctic Studies, University of Tasmania, Hobart, TAS 7005, Australia
[5] School of Life Sciences, University of Warwick, Coventry CV4 7AL, UK
* Correspondence: suhn@sdu.edu.cn (H.S.); yqzh1989@sdu.edu.cn (Y.Z.);
Tel.: +86-532-58632590 (H.S.); +86-532-58632576 (Y.Z.)

Table S1 Bacterial strains and plasmids used in this study.

Strain or plasmid	Description[a]	Source
Strains		
SM9913	Wild type	[26]
Δ*filZ*	*filZ* gene deletion mutant of SM9913	This study
Δ*2230*	*PSM_A2230* gene deletion mutant of SM9913	This study
Δ*filZ*Δ*2230*	Deletion mutant of the DNA region containing *filZ* and *PSM_A2230* genes	This study
Δ*0915*	*PSM_A0915* gene deletion mutant of SM9913	This study
9913(pEV)	Wild type strain harboring pEV plasmid	This study
Δ*filZ*(pEV)	Δ*filZ* mutant strain harboring pEV plasmid	This study
Δ*filZ*(pEV^{filZ})	Complemented strain of the Δ*filZ* mutant	This study
Δ*filZ*(pEV$^{filZ\text{-}R13A}$)	Δ*filZ* mutant strain expressing the mutant protein FilZ-R13A	This study
Δ*0915*(pEV^{0915})	Complemented strain of the Δ*0915* mutant	This study
Δ*0915*(pEV^{filZ})	Δ*0915* mutant strain expressing protein FilZ	This study
Δ*0915*(pEV$^{filZ\text{-}R13A}$)	Δ*0915* mutant strain expressing protein FilZ-R13A	This study
Δ*0915*(pEV)	Δ*0915* mutant strain harboring pEV plasmid	This study
E. coli MW3064	Donor strain for conjugation	[54]
E. coli DH5α	Strain for cloning	Vazyme
E. coli BL21(DE3)	Strain for high level expression of heterologous proteins	Vazyme
Plasmids		
pK18-*mobsacB*-Ery	Suicide vector for gene knockout, Kan^r, Ery^r	[54]
pK18-*filZ*	pK18*mobsacB*-Ery containing the homologous arms of *filZ* gene	This study
pK18-*2230*	pK18*mobsacB*-Ery containing the homologous arms of *PSM_A2230* gene	This study
pEV	Cloning vector for gene complementation, Amp^r, Cm^r	[38]
pEV^{filZ}	pEV plasmid expressing FilZ	This study
pEV$^{filZ\text{-}R13A}$	pEV plasmid expressing the mutant protein FilZ-R13A	This study
pEV^{0915}	pEV plasmid expressing protein A0915	This study
pKT25	The constructed plasmid in two-hybrid system	[35]
pUT18C	The constructed plasmid in two-hybrid system	[35]
pKT25-*2230*	The constructed plasmid in two-hybrid system, expressing the fusion protein T25-A2230	This study
pKT25-*cheA*	The constructed plasmid in two-hybrid system, expressing the fusion protein T25-CheA	This study
pKT25-*fliM$_p$*	The constructed plasmid in two-hybrid system, expressing the fusion protein T25-FliM$_p$	This study
pKT25-*fliM*	The constructed plasmid in two-hybrid system, expressing the fusion protein T25-FliM	This study
pUT18C-*2230*	The constructed plasmid in two-hybrid system, expressing the fusion protein T18-A2230	This study
pUT18C-*cheY*	The constructed plasmid in two-hybrid system, expressing the fusion protein T18-CheY	This study
pUT18C-*filZ*	The constructed plasmid in two-hybrid system, expressing the fusion protein T18-FilZ	This study
pUT18C-*filZ-R13A*	The constructed plasmid in two-hybrid system, expressing the fusion protein T18-FilZ-R13A	This study
pET22b-*filZ*	Expressing protein FilZ	This study
pET22b-*A2230*	Expressing protein A2230 with a C-terminal 6x His tag	This study
pGEX4T-1-*filZ*	Expressing protein FilZ with an N-terminal GST tag	This study

pGEX4T-1-*filZ-R13A*	Expressing protein FilZ-R13A with an N-terminal GST tag	This study

[a]Amp^r, ampicillin resistance; Ery^r, erythromycin resistance; Kan^r, kanamycin resistance; Cm^r, chloramphenicol resistance.

Table 2 Primers used in this study[a].

Primer (s)	Sequence (s) (5'–3')
For two-hybrid assay	
pKT25-*cheA*-F/R	GACTCTAGAGGATCCCCGGATGAGCTTTGAAGTCGATGA/CGAATTCTTAGTTACTTAGGTACTTATCCAGCGTAATGCTTTA
pKT25-*fliM$_p$*-F/R	GACTCTAGAGGATCCCCGGGTGAGCGATTTATTATCCCA/CGAATTCTTAGTTACTTAGGTACTTACCAGTCTAAGTCTACAC
pKT25-*fliM*-F/R	GACTCTAGAGGATCCCCGGATGAGTAAAACGTTAAGCGC/CGAATTCTTAGTTACTTAGGTACTTAATTACTCGTTGTATTGT
pKT25-*2230*-F/R	GACTCTAGAGGATCCCCGGATGAGTGAAGATAAATTACT/CGAATTCTTAGTTACTTAGGTACTTATATATCGCTTAACTCAT
pUT18C-*filZ*-F/R	GACTCTAGAGGATCCCCGGATGAGTAACCAATCAGAAAC/ACTTAGTTATATCGATGAATTCGACTATGCATTGGTTTTTTTG
pUT18C-*2230*-F/R	GACTCTAGAGGATCCCCGGATGAGTGAAGATAAATTACT/ACTTAGTTATATCGATGAATTCGATTATATATCGCTTAACTCAT
pUT18C-*cheY*-F/R	GACTCTAGAGGATCCCCGGTTGGATAAAAACATTAAAAT/ACTTAGTTATATCGATGAATTCGATTAACCTAAGCGTTCGAACA
pUT18C-*filZ-R13A*-F/R	ATCTTTGAGGTTTAAATTCCATGCTTTGTAACTTCGTTTAGTTTCTGATTGGTTAC/GTAACCAATCAGAAACTAAACGAAGTTACAAAGCATGGAATTTAAACCTCAAAGAT
For gene mutation and complementation	
pK18-*filZ*-up-F/R	GTAAAACGACGGCCAGTGCCAAGCTTAATGATGGAAAACAAAAATC/AATAGCCTTTTCAGCAGGCATTACTCATAATTCTCTTCAA
pK18-*filZ*-down-F/R	TTGAAGAGAATTATGAGTAATGCCTGCTGAAAAGGCTATT/GTCATAAGATTAGTCACTGGGGATCCGCTTTTAGTTGTTTTAATGT
pK18-*2230*-up-F/R	GTAAAACGACGGCCAGTGCCAAGCTTACTTTGATTATGCCGAGCTG/GACCATTACGAGGTAAGAGCGCTTCTCTACATAAAACTAT
pK18-*2230*-down-F/R	ATAGTTTTATGTAGAGAAGCGCTCTTACCTCGTAATGGTC/GTCATAAGATTAGTCACTGGGGATCCAACCTCTCAAGCGAAAAGCT
pK18-*0915*-up-F/R	GTAAAACGACGGCCAGTGCCAAGCTTGTGCATTATTTTTGACGCAA/CATTTTATTTTTCCTTCAAAGAGTTCCGGTTTACTAATAA
pK18-*0915*-down-F/R	TTATTAGTAAACCGGAACTCTTTGAAGGAAAAATAAAATG/GTCATAAGATTAGTCACTGGGGATCCTCAAGCCCTGGCACACTGCC
filZ-screen-F/R	AAAAGTGATGACCATGGGCG/AATGCGAACAAGGCGAAATTTC
2230-screen-F/R	ACCAGCTCTTGGTTTTGTTGTT/CATCTTTAAATTATCAATATCTCATCACTTTAGGT
0915-screen-F/R	CGCTACCCCATTATTAAAAATTCAAAG/ACTGGCAATCATCGCCCC

pEV^{filZ}-F/R	TAAAAAACACACAACAGGAGATCTATGAGTAACCAATCAGAAAC/GGTGGTGGTGGTGCTCGAGCTATGCATTGGTTTTTTTG
pEV$^{filZ-R13A}$-F/R	ATCTTTGAGGTTTAAATTCCATGCTTTGTAACTTCGTTTAGTTTCTGATTGGTTAC/GTAACCAATCAGAAACTAAACGAAGTTACAAAGCATGGAATTTAAACCTCAAAGAT
pEV^{0915}-F/R	TAAAAAACACACAACAGGAGATCTGTGTTTGTTGATGCTGATAC/GGTGGTGGTGGTGCTCGAGTTATGCAATTGCATCAATAT
For heterologous protein expression	
pET22b-*filZ*-F/R	TAAGAAGGAGATATACATATGATGAGTAACCAATCAGAAAC/GTGGTGGTGGTGGTGCTCGAGCTATGCATTGGTTTTTTTG
pGEX4T-1-*filZ*-F/R	GGATCTGGTTCCGCGTGGATCCATGAGTAACCAATCAGAAAC/ACGATGCGGCCGCTCGAGCTATGCATTGGTTTTTTTG
pGEX4T-1-*filZ-R13A*-F/R	ATCTTTGAGGTTTAAATTCCATGCTTTGTAACTTCGTTTAGTTTCTGATTGGTTAC/GTAACCAATCAGAAACTAAACGAAGTTACAAAGCATGGAATTTAAACCTCAAAGAT
pET22b-*A2230*-F/R	TAAGAAGGAGATATACATATGATGAGTGAAGATAAATTACT/GTGGTGGTGGTGGTGCTCGAGTTATATATCGCTTAACTCAT
For real-time qPCR	
rpoD-rt-F/R	CGCATATTATTGACTGGTTAGGTG/CAAGGGTTGAGGGTTCATAGC
filZ-rt-F/R	ATTACGAGCCAGAGTCAGTTCAG/CCACACCACCCACGCTAATG
2230-rt-F/R	CCTTCGTATGTGATTGGTAT/CTTGCTCTTCTGCTTCAA

[a]The underlined nucleotides represent restriction sites.

Table S3 (separate file).

Table S3 is available as a separate Excel file. The legend is given below.

Table S3 Information of bacteria involved in the phylogenetic analysis of FilZ homologs and the alignment of the lateral flagellar cluster regions containing the *filZ* gene. NA, not available.

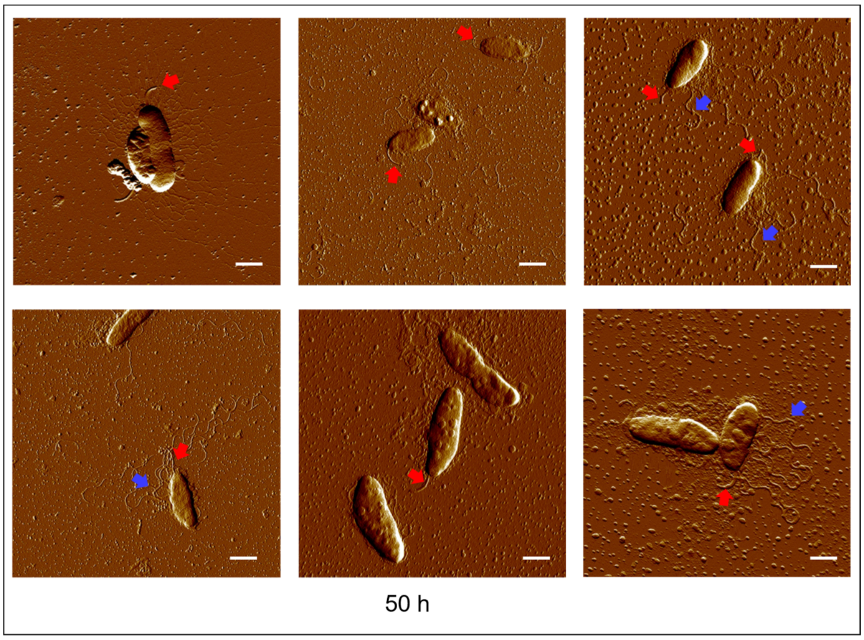

Figure S1. Observation of the production of flagella during strain SM9913 swarming at 50 h. Cells were extracted from the edge of the swarming colonies. The red arrow points to the polar flagellum, and the blue arrow points to the lateral flagella. Bar, 1 μm.

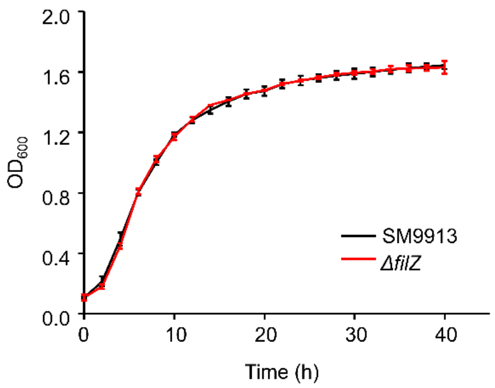

Figure S2. Growth curves of strain SM9913 and its mutant *ΔfilZ*. The strains were cultivated in marine LB broth at 15°C. The graph shows data from triplicate experiments (mean ± SD).

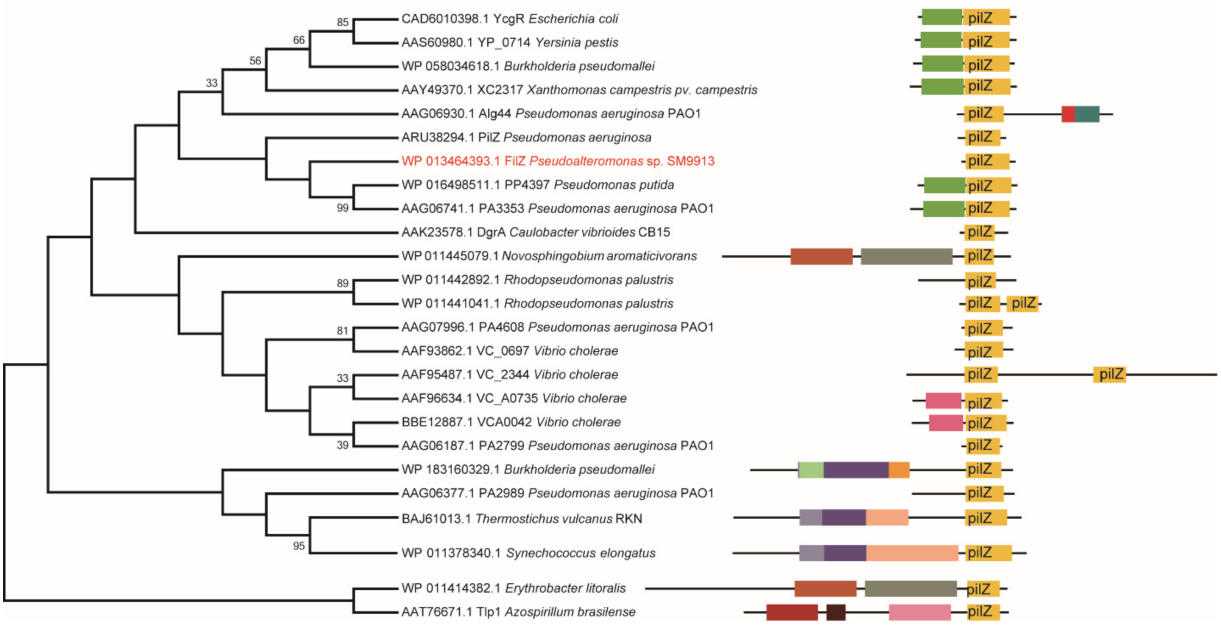

Figure S3. Phylogenetic analysis of bacterial PilZ domains. The left cladogram shows evolutionary relationships between PilZ domains inferred from maximum parsimony analysis of a multiple sequence alignment. The right diagrams schematize the domain organization of each PilZ protein inferred using RPS-BLAST analysis of the Conserved Domain Database (https://www.ncbi.nlm.nih.gov/Structure/cdd). FilZ of strain SM9913 was marked in red.

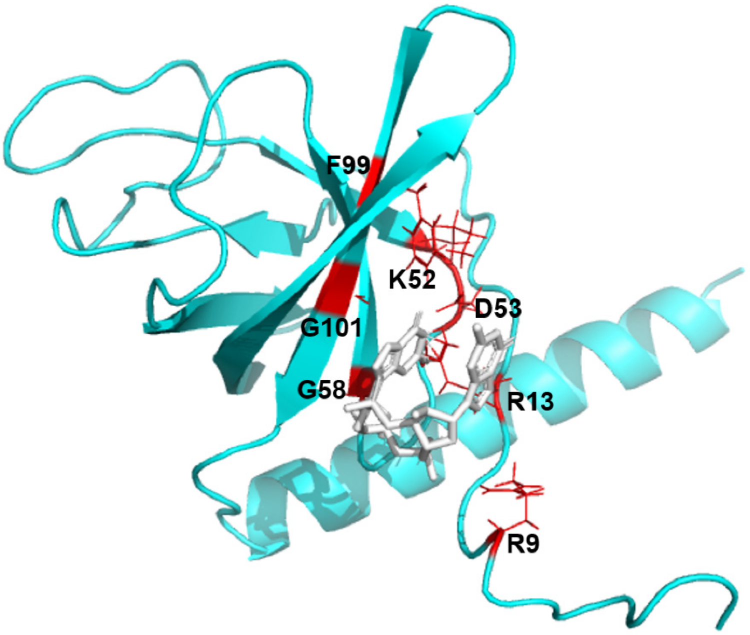

Figure S4. Predicted structure of protein FilZ (cyan) complexed with the c-di-GMP molecule (grey). The structure of FilZ was predicted by using I-Tasser (https://seq2fun.dcmb.med.umich.edu/I-TASSER-MR/) based on the structure of the C-terminal PilZ domain of protein YcgR (YcgR-PilZ) from *E. coli* (PDB ID 5Y6F). The possible residues of FilZ involved in binding c-di-GMP, including R9, R13, K52, D53, G58, F99 and G101, are marked in red.

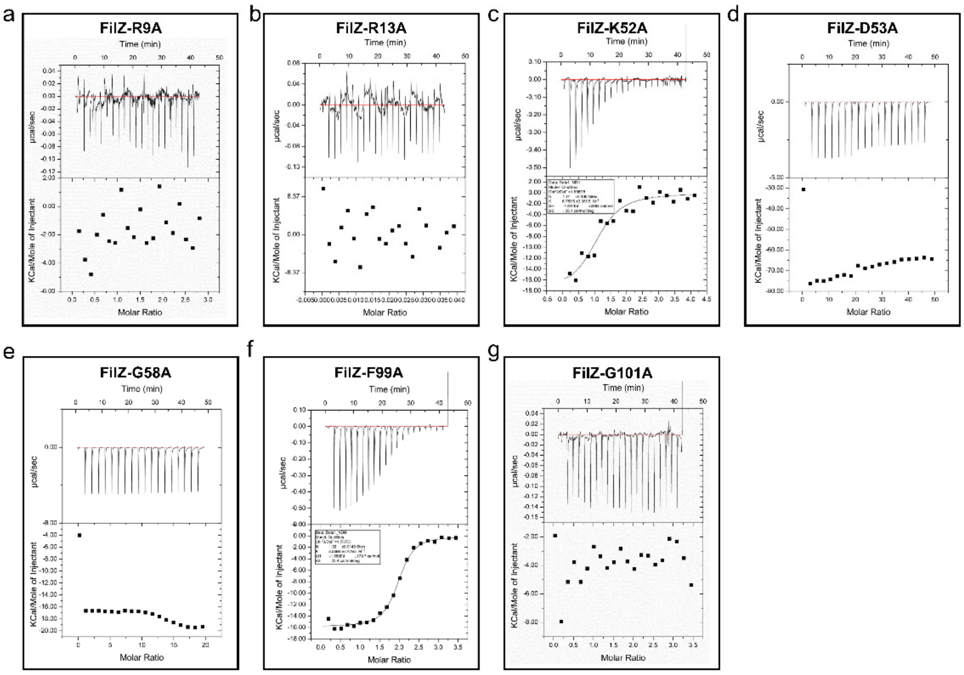

Figure S5. ITC detection of the c-di-GMP binding ability of recombinant FilZ mutants including FilZ-R9A (a), FilZ-R13A (b), FilZ-K52A (c), FilZ-D53A (d), FilZ-G58A (e), FilZ-F99A (f) and FilZ-G101A (g). The upper panels show the raw calorimetric data of the titration, and the lower panels show the corresponding integrated injection heats. The curves represent the best least-squares fits to the one binding site model. The graphs are representatives of at least three replicates.

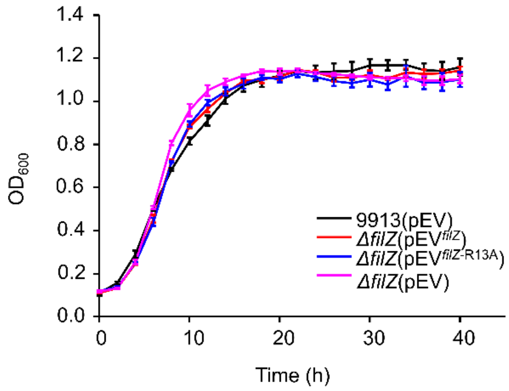

Figure S6. Growth curves of strains 9913(pEV), *ΔfilZ*(pEV^{filZ}), *ΔfilZ*(pEV$^{filZ\text{-}R13A}$) and *ΔfilZ*(pEV). All these strains were cultivated in marine LB broth at 15°C. The graph shows data from triplicate experiments (mean ± SD).

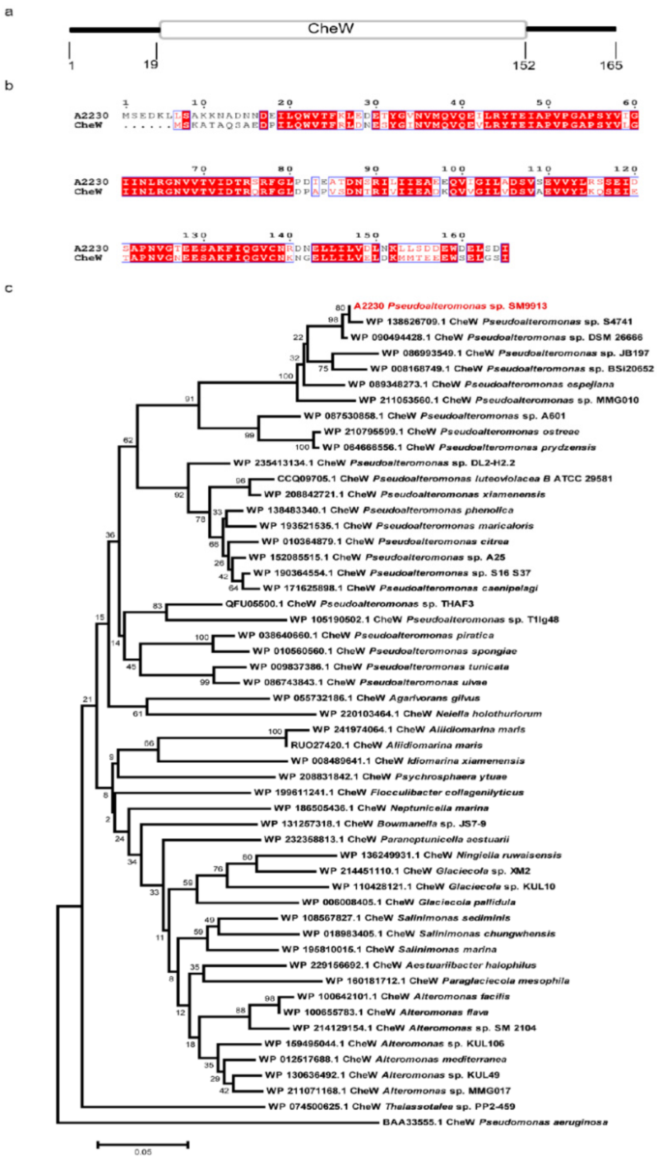

Figure S7. Sequence analysis of protein A2230. (a) Prediction of the protein domain in A2230 using the NCBI conserved domain database (https://www.ncbi.nlm.nih.gov/Structure/cdd). (b) Sequence alignment between protein A2230 and protein CheW (GenBank: BAA33555.1) of Pseudomonas aeruginosa. Sequence alignment shows a high identity (69%, 110/159 residues) between the two proteins. (c) Phylogenetic analysis of A2230 and its homologs obtained from the GenBank database by using MEGA 5. The phylogenetic tree was constructed using the neighbor-joining method with 1000 bootstrap replicates. The numbers at each tree node are the bootstrap values.

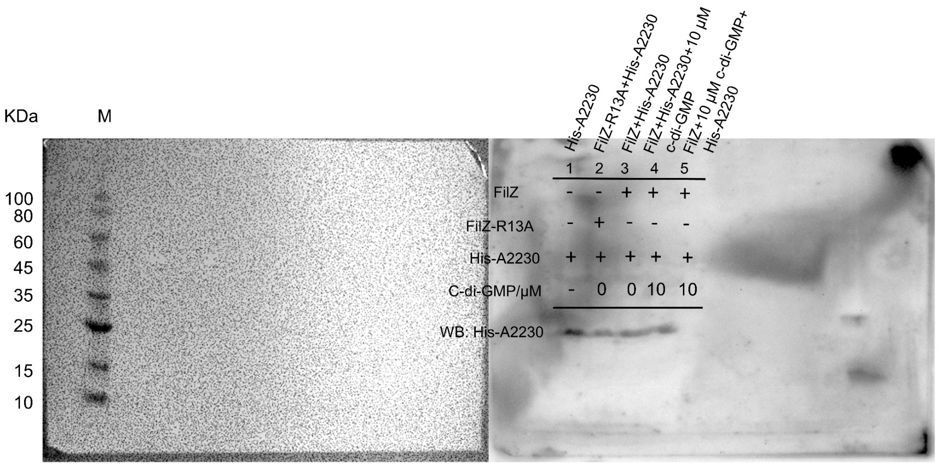

Figure S8. Pull down assay of the interaction between FilZ or FilZ-R13A and His-A2230 *in vitro* combined with Western Blot. The protein marker (the left) and the protein bands (the right) were from the same membrane of the Western Blot assay. Lanes 1 to 5, His-A2230 only, the mixture of FilZ-R13A and His-A2230, the mixture of FilZ and His-A2230, the addition of 10 μM c-di-GMP in the FilZ-A2230 mixture, and addition of A2230 in the FilZ-c-di-GMP mixture, respectively.

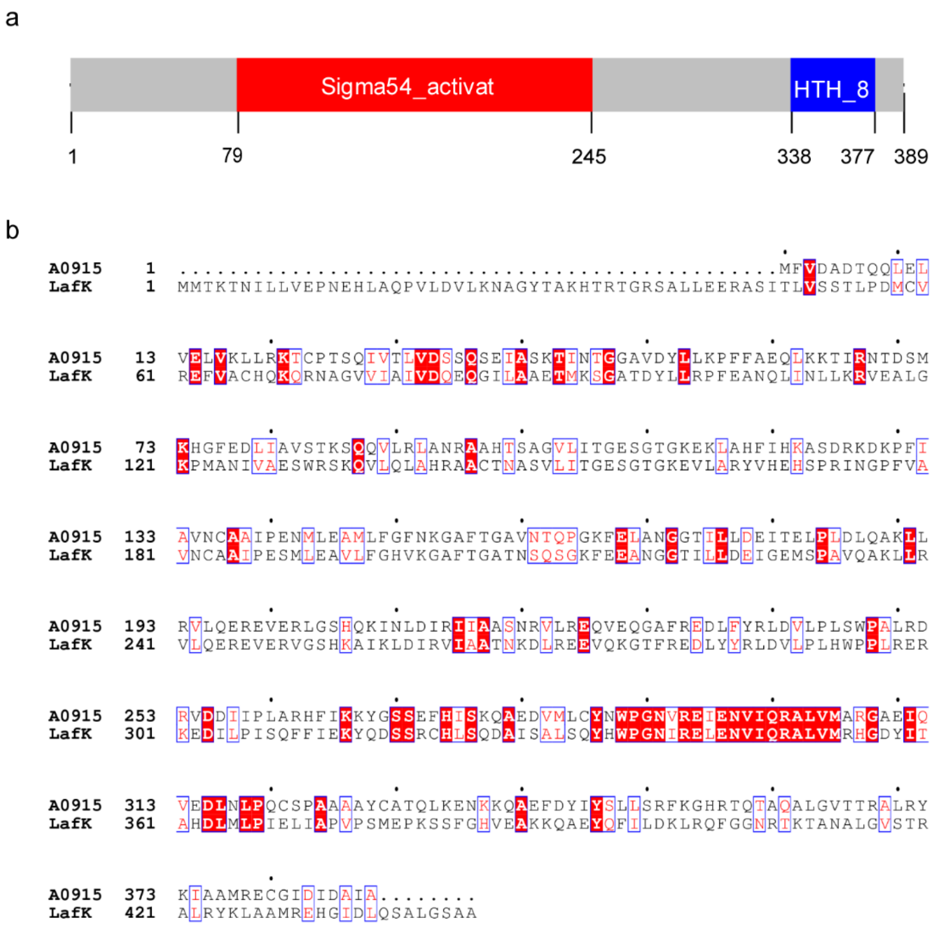

Figure S9. Sequence analysis of protein A0915. (a) Prediction of the protein domain in A0915 using the NCBI conserved domain database (https://www.ncbi.nlm.nih.gov/Structure/cdd). Protein A0915 contains an N-terminal Sigma54_activat domain (pfam00158, E-value 5.72e-102) and a C-terminal HTH_8 domain (pfam02954, 5.11e-04). (b) Sequence alignment between protein A0915 and protein LafK (GenBank: SUQ08311.1) of *Vibrio parahaemolyticus*. Sequence alignment shows a high identity (40.9%, 211/378 residues) between the two proteins.
